# A reply to “Addressing gender imbalance in intensive care”

**DOI:** 10.1186/s13054-021-03601-w

**Published:** 2021-06-22

**Authors:** Federica Fusina

**Affiliations:** Department of Anesthesia, Intensive Care and Pain Medicine, Fondazione Poliambulanza Hospital, Via Bissolati, 57, 25124 Brescia, Italy

**Dear Editors,**

As an intensive care physician, a researcher, a woman, and a mother of two small children, I read with great interest the viewpoint by Professor J.L. Vincent and colleagues, entitled “Addressing gender imbalance in intensive care” [1].

I completely agree on the fact that balancing medicine and family is a challenge, and the perception of “needing to choose” between work or family is still strong for female intensivists. Breastfeeding facilities, childcare policies, and institutions that promote flexibility are still lacking in many parts of the world and, as noted, are essential for reaching gender equity [2].

However, I think that strategies such as “giving grants and awards alternately to a male and female intensivist” and “ensuring gender balance in committees and as speakers at conferences and scientific events (applying quota if deemed appropriate)” are far from what we need to achieve gender balance in the workplace. Awards and grants should be given to those who deserve them, and speakers should be chosen on their scientific merits and abilities, and not on their gender. I would find it offensive to know I have been chosen for a role just for my gender, and I believe many of my colleagues, both male and female, share this point of view.

What we really need is a cultural change. I think that we will achieve a true gender balance in intensive care when parenthood will become a responsibility that is shared equally between both parents. An important step for reaching this goal is requiring men to take paternity leave, as you suggested, but I believe that a shift in the language we regularly use is no less important.

For example, the authors suggest that a method to improve gender balance could be to “provide conditions like maternity leave and in‐hospital nursery schools in order to facilitate female intensivists during their early motherhood period”.

I think that if we truly wish to reach our goal, in the future this should be reworded as “provide conditions like maternity or paternity leave and in‐hospital nursery schools in order to facilitate intensivists during their early parenthood period”.

I wish to thank the authors for reflecting on this very important aspect and hope that a true gender balance in intensive care is not far from becoming reality.

## Data Availability

Not applicable.
