# Supplementary material for: A reply to “Addressing gender imbalance in intensive care”
Source: Crit Care. 2021 Jun 22;25:216. doi: 10.1186/s13054-021-03601-w (PMC8220742; doi:10.1186/s13054-021-03601-w)
Supplement: Related texts

# Reply: Authors’ response

**Authors:** Francesca Rubulotta, Karen E. A. Burns, V. Marco Ranieri, Nicole P. Juffermans, Chryssa Pourzitaki, Jean-Louis Vincent

**Affiliations:** 2 grid.7445.2 0000 0001 2113 8111 Department of Anaesthesia and Intensive Care Medicine, Imperial College London, London, UK; 3 Chair of the International Women in Intensive and Critical Care Medicine Network, Catania, Italy; 4 grid.17063.33 0000 0001 2157 2938 Interdepartmental Division of Critical Care Medicine, University of Toronto, Toronto, ON Canada; 5 grid.415502.7 Unity Health Toronto-St. Michael’s Hospital, Toronto, ON Canada; 6 grid.415502.7 Li Ka Shing Knowledge Institute, St. Michael’s Hospital, Toronto, ON Canada; 7 grid.6292.f 0000 0004 1757 1758 Dipartimento Di Scienze Mediche E Chirurgiche, Anesthesia and Intensive Care Medicine, Alma Mater Studiorum, Università Di Bologna, Policlinico Di Sant’Orsola, Bologna, Italy; 8 grid.5650.6 0000000404654431 Laboratory of Experimental Intensive Care and Anaesthesiology, Amsterdam University Medical Centre, Location Academic Medical Centre, Amsterdam, The Netherlands; 9 grid.440209.b 0000 0004 0501 8269 Department of Intensive Care, OLVG Hospital, Amsterdam, The Netherlands; 10 grid.4793.9 0000000109457005 Department of Anesthesiology and ICU, Faculty of Medicine, School of Health Sciences, Aristotle University of Thessaloniki, 54124 Thessaloniki, Greece; 11 grid.4989.c 0000 0001 2348 0746 Department of Intensive Care, Erasme Hospital, Université Libre de Bruxelles, Brussels, Belgium

The authors thank Dr. Fusina for her comments and for the opportunity to further clarify some of the issues raised in our viewpoint [1]. We fully agree that scientific merit and ability must take priority before any consideration of gender, and that language is important. An approach is needed that ensures that recruitment, rewards, and promotion are designed in ways that minimize any potential for gender bias, in either direction. For example, job advertisements and role descriptions could be reviewed for gendered language; recruitment directors and organizers of academic events could receive formal training to limit unconscious biases [3]; professional organizations (including academic), scientific societies, and congress committees could be encouraged to regularly report diversity metrics pertaining to the number of shortlisted candidates, faculty, awards, and appointments, as these data enhance awareness of the importance of diversity and provide useful benchmarks.

As Dr. Fusina highlights, an important move towards changing culture and challenging gender stereotypes is to facilitate greater equity in parenthood and care roles, actively and equally supporting men and women who wish to take time from work to care for children, elderly parents, or other dependants. To this end, the UK Government Equalities Office’s guidance document for employers promotes effective action to narrow the gender pay gap and provides increased payment for shared parental leave [4]. The British Medical Association’s Junior Doctor Committee successfully campaigned to secure enhanced pay for shared parental leave in the National Health System (NHS), equivalent to the enhanced maternity pay entitlement. Similarly, the Stanford/Kaiser Emergency Medicine Residency Program, USA, established a new policy to facilitate return-to-work for new resident parents, both mothers and fathers [5]. As more men take on these roles, current workplace culture and gender stereotypes will gradually be eroded, helping shape a more inclusive society in the future.

Many changes, included those mentioned herein, are needed to breakdown structural and cultural barriers to gender balance in workplaces and society. Although the number of women in leadership and academic roles will gradually and spontaneously increase because of current trends, we should not rely on this passive transformation, but must all actively and determinedly participate in promoting change until gender equity is established as the norm across all sections of society, including critical care medicine.
